# Combined negative pressure wound therapy with irrigation and dwell time and artificial dermis prevents infection and promotes granulation formation in a ruptured giant omphalocele: a case report

**DOI:** 10.1186/s12887-022-03755-8

**Published:** 2022-11-26

**Authors:** Yoichi Nakagawa, Hiroo Uchida, Akinari Hinoki, Chiyoe Shirota, Wataru Sumida, Satoshi Makita, Hizuru Amano, Masamune Okamoto, Aitaro Takimoto, Seiya Ogata, Shunya Takada, Daiki Kato, Yousuke Gohda

**Affiliations:** 1 Department of Pediatric Surgery, Nagoya University Graduate School of Medicine, 65 Tsurumai-cho, Showa-ku, 466-8550 Nagoya, Aichi Japan; 2 Department of Rare/Intractable Cancer Analysis Research, Nagoya University Graduate School of Medicine, 65 Tsurumai-cho, Showa-ku, 466-8550 Nagoya, Japan

**Keywords:** Negative pressure wound therapy, Negative pressure wound therapy with irrigation and dwell time, Omphalocele

## Abstract

**Background:**

Omphalocele is a congenital abdominal wall defect of the umbilical cord insertion site. A giant omphalocele, with a fascial defect > 5 cm in diameter and/or containing > 50% of the liver within the hernia sac, can be challenging for pediatric surgeons. Recently, negative pressure wound therapy has been reported as an effective management for giant omphaloceles; however, it is not recommended for an infected wound with necrotic tissue as it may exacerbate infection. We adopted negative pressure wound therapy with irrigation and dwell time (NPWTi-d) for a case of a ruptured giant omphalocele. Artificial membranes, followed by artificial dermis, were used to promote fibrous capsule formation, and then NPWTi-d was used to promote granulation while controlling infection. However, studies have not been conducted regarding NPWTi-d for ruptured giant omphaloceles; hence, we present our treatment experience with NPWTi-d for a giant omphalocele.

**Case presentation:**

The patient was a boy born at 38 weeks and 3 days of gestation, weighing 1896 g. He was diagnosed with a ruptured giant omphalocele with a total liver and intestine defect hole of 10 cm × 10 cm. The patient underwent silo placement using an artificial mesh, followed by plicating the artificial mesh at 4 days of age. The herniated viscera were gradually reduced into the abdominal cavity; however, the defect size was still large. Hence, a collagen-based artificial dermis was patched on the defect hole. After creating a fresh and smooth granulated tissue, NPWTi-d was applied at 33 days of age to promote granulation and control infection. We used the 3 M™ V.A.C.® Ulta Therapy Unit with 3 M™ VeraFlo™ therapy. NPWTi-d was stopped at 60 days of age when the granulation tissue was well formed including at the artificial dermis site. The wound was managed with prostandin ointment and appropriate debridement, resulting in complete epithelialization at 5 months of age.

**Conclusions:**

Artificial membranes followed by artificial dermis were used to promote a fibrous capsule and artificial dermis granulation, which protects against organ damage. NPWTi-d achieved better control of infection and promoted wound healing. NPWTi-d combined with artificial dermis can effectively treat ruptured giant omphaloceles.

## Background

An omphalocele is a congenital abdominal wall defect at the umbilical cord insertion site. In embryology, the lateral and craniocaudal abdominal folds close and form the umbilical ring, and the herniated midgut returns into the body cavity at the 12th week of gestation [1]. Omphalocele is thought to be caused by the arrest of the abdominal wall folding *in utero* [2].

Treating giant omphaloceles can be challenging for pediatric surgeons. No consensus has been established on the strict definition of the term “giant,” although some reports define omphaloceles having a fascial defect > 5 cm in diameter and/or that contains > 50% of the liver within the hernia sac as giant omphaloceles [3, 4].

Recently, negative pressure wound therapy (NPWT) has been reported as an effective management strategy for giant omphaloceles [5–7]. NPWT is a useful treatment; however, it is only indicated for wounds with controlled infection in the package insert. An infected wound with necrotic tissue is not recommended for NPWT because of the exaggeration of infection.

We encountered a case of ruptured giant omphalocele that was infected during silo placement. To control infection and promote granulation tissue formation, we adopted NPWT with irrigation and dwell time (NPWTi-d). We have routinely introduced NPWTi-d for the initial management of omphalocele and gastroschisis since 2019. We combined NPWTi-d and artificial patches to achieve good granulation formation and epithelialization. Artificial membranes, followed by artificial dermis, were used to first promote fibrous capsule formation, and then NPWTi-d was used to promote granulation while controlling infection. However, studies regarding NPWTi-d for a ruptured giant omphalocele have not been conducted to the best of our knowledge. Hence, we present our treatment experience of NPWTi-d for a giant omphalocele.

## Case presentation

**Fig. 1:**
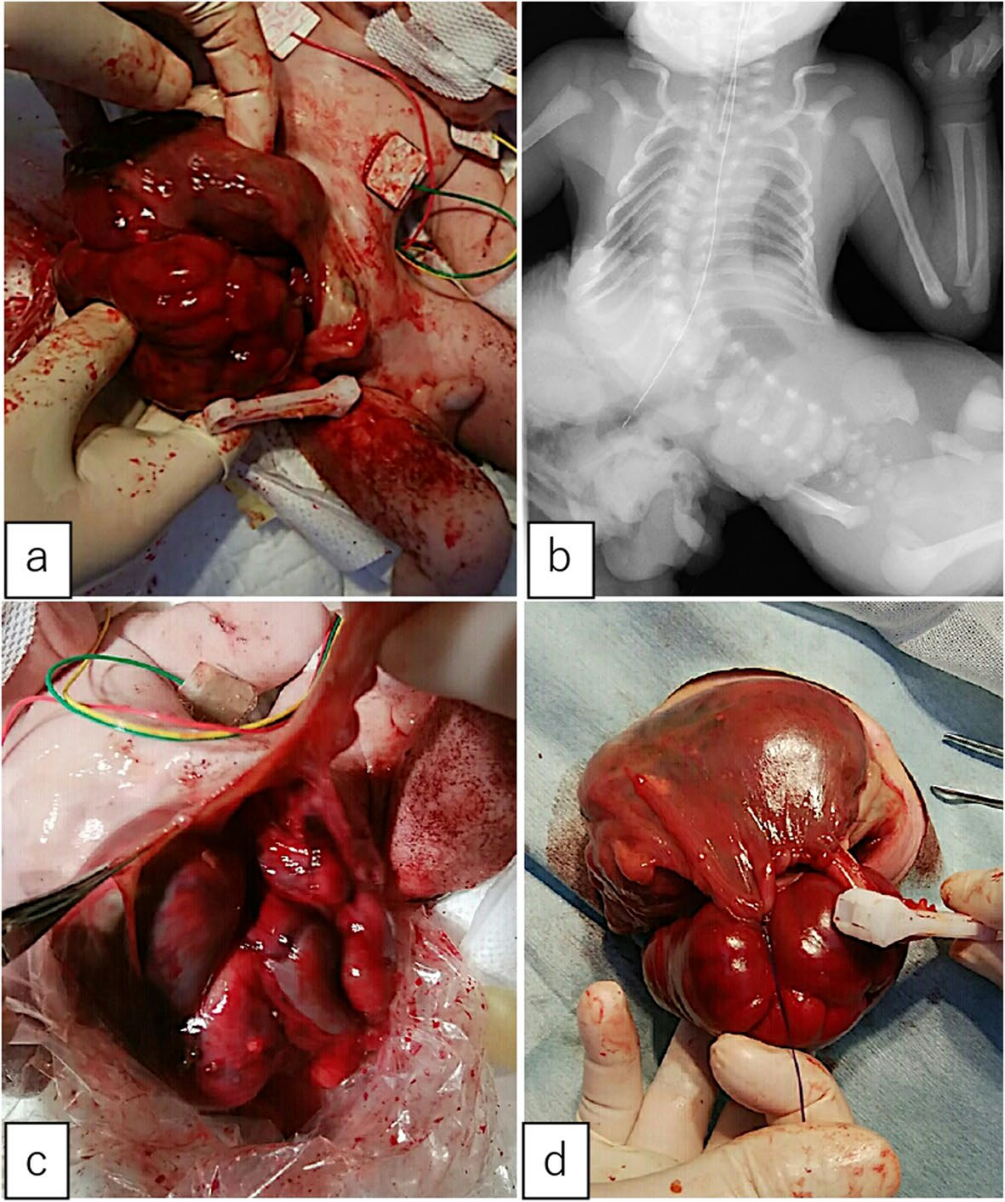
** a** A ruptured giant omphalocele. The defect hole was 10 cm × 10 cm, with herniation of the liver and total intestine. **b** The X-ray reveals scoliosis. **c**, **d** The ruptured membrane did not cover the defect hole

**Fig. 2:**
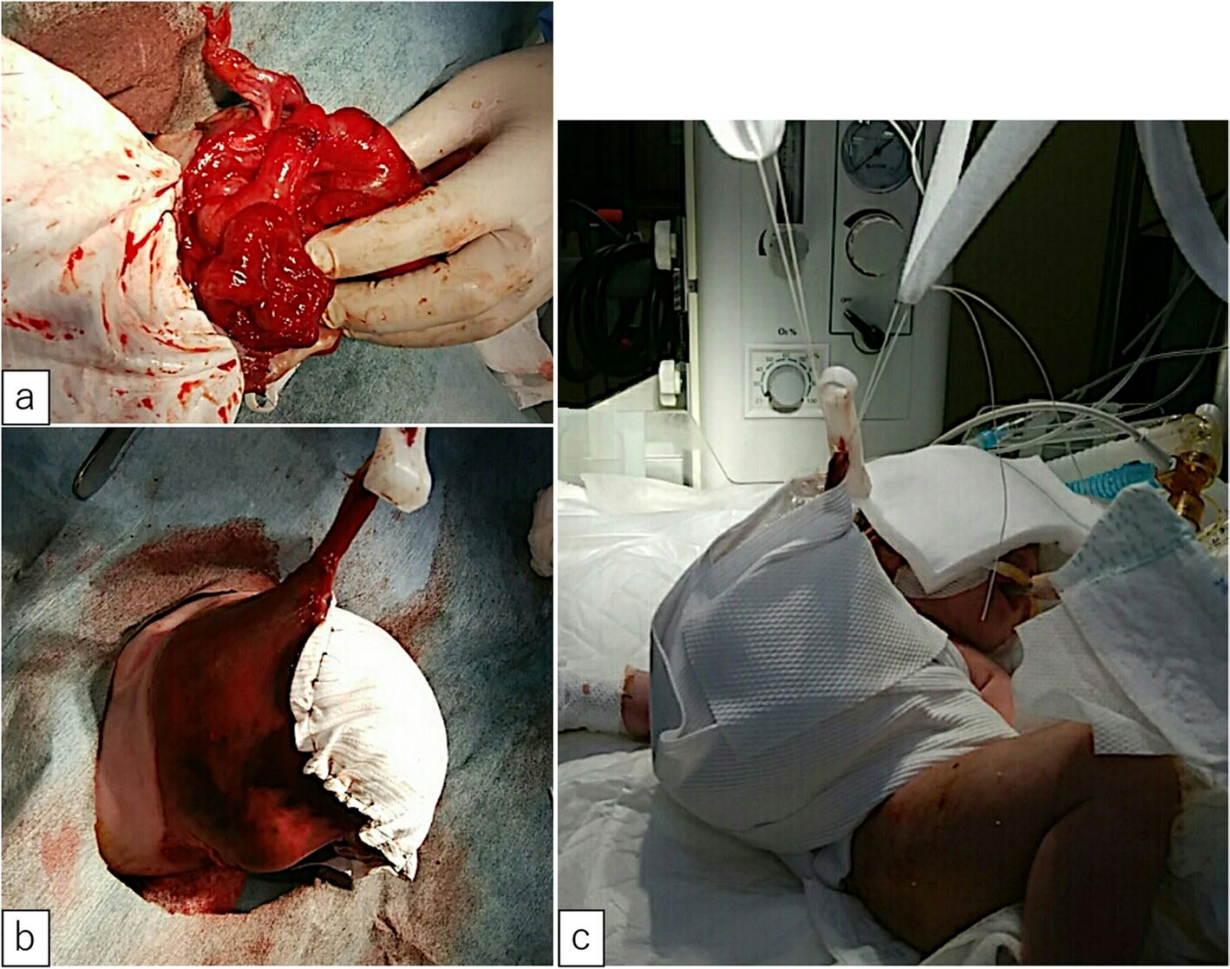
** a**, **b** GORE® DUALMESH® Biomaterial was sutured with the remaining amniotic membrane and skin. **c** Silo placement

**Fig. 3:**
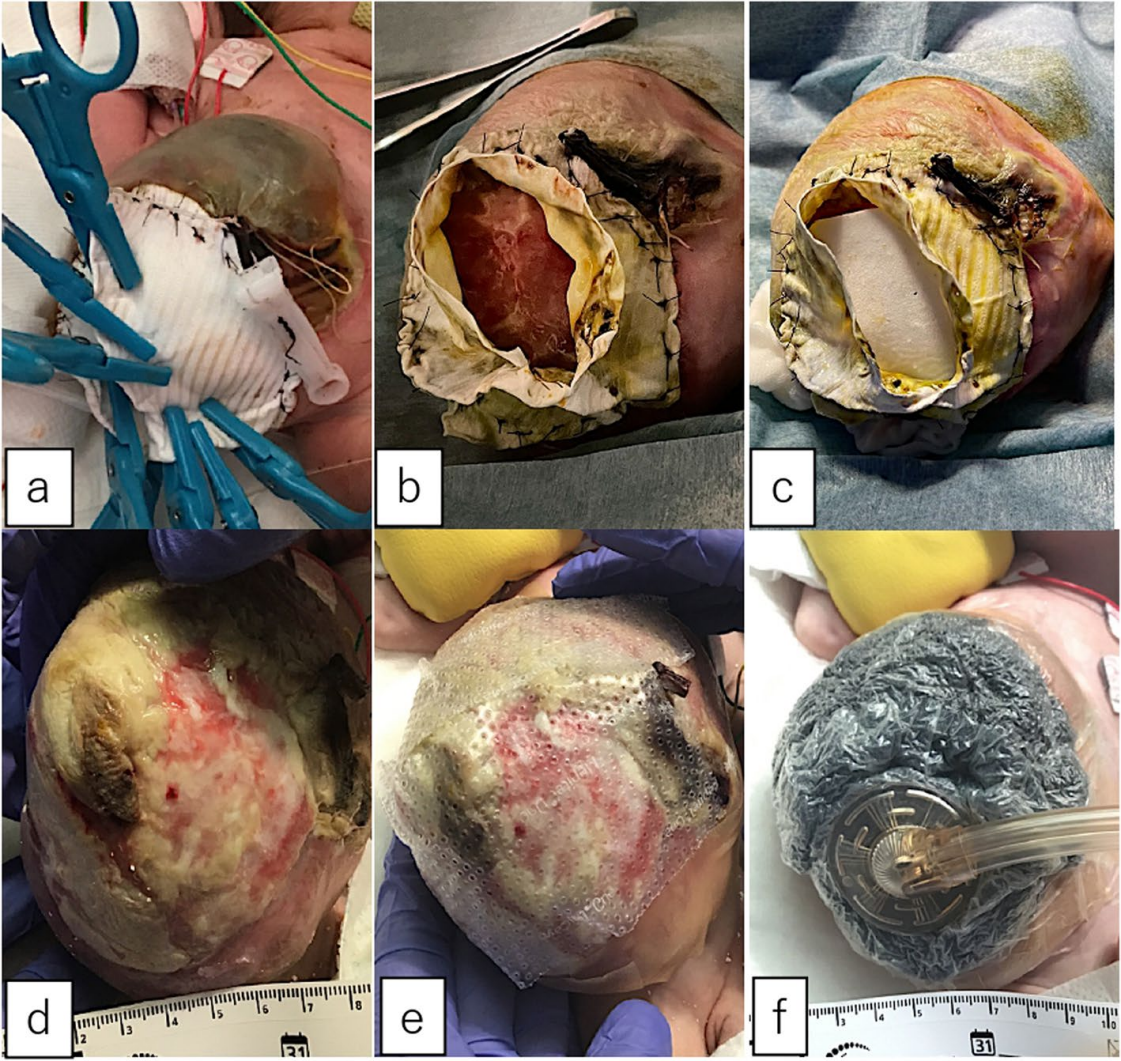
** a** The artificial mesh was plicated to reduce the herniation of the organs. **b** The defect hole was covered with a fibrous capsule. **c** A TERUDERMIS® artificial dermis silicone membrane type covered the defect hole.  **d** A fresh and smooth granulate tissue was created at the defect hole. **e** Mepitel® One was used to cover the fragile wound site. **f** V.A.C. Veraflo™ Therapy Dressing was set above the Mepitel® One

**Fig. 4:**
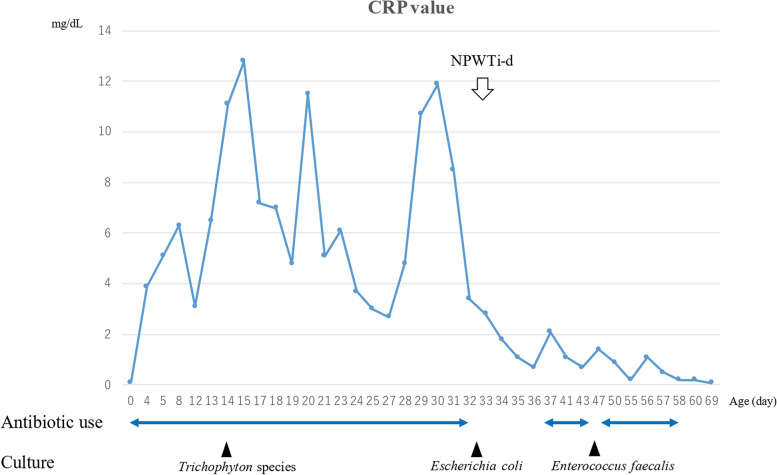
Graph chart of changes in the inflammatory marker, i.e. CRP (C-reactive protein). Antibiotic was used during 0 − 31, 37 − 43, and 48 − 57 days of age. Wound culture was positive for *Trichophyton* species, *Escherichia coli*, and *Enterococcus faecalis*, at the age of 14, 33, and 48 days, respectively. NPWTi-d (negative pressure wound therapy with irrigation and dwell time) was started at the age of 33 days

**Fig. 5:**
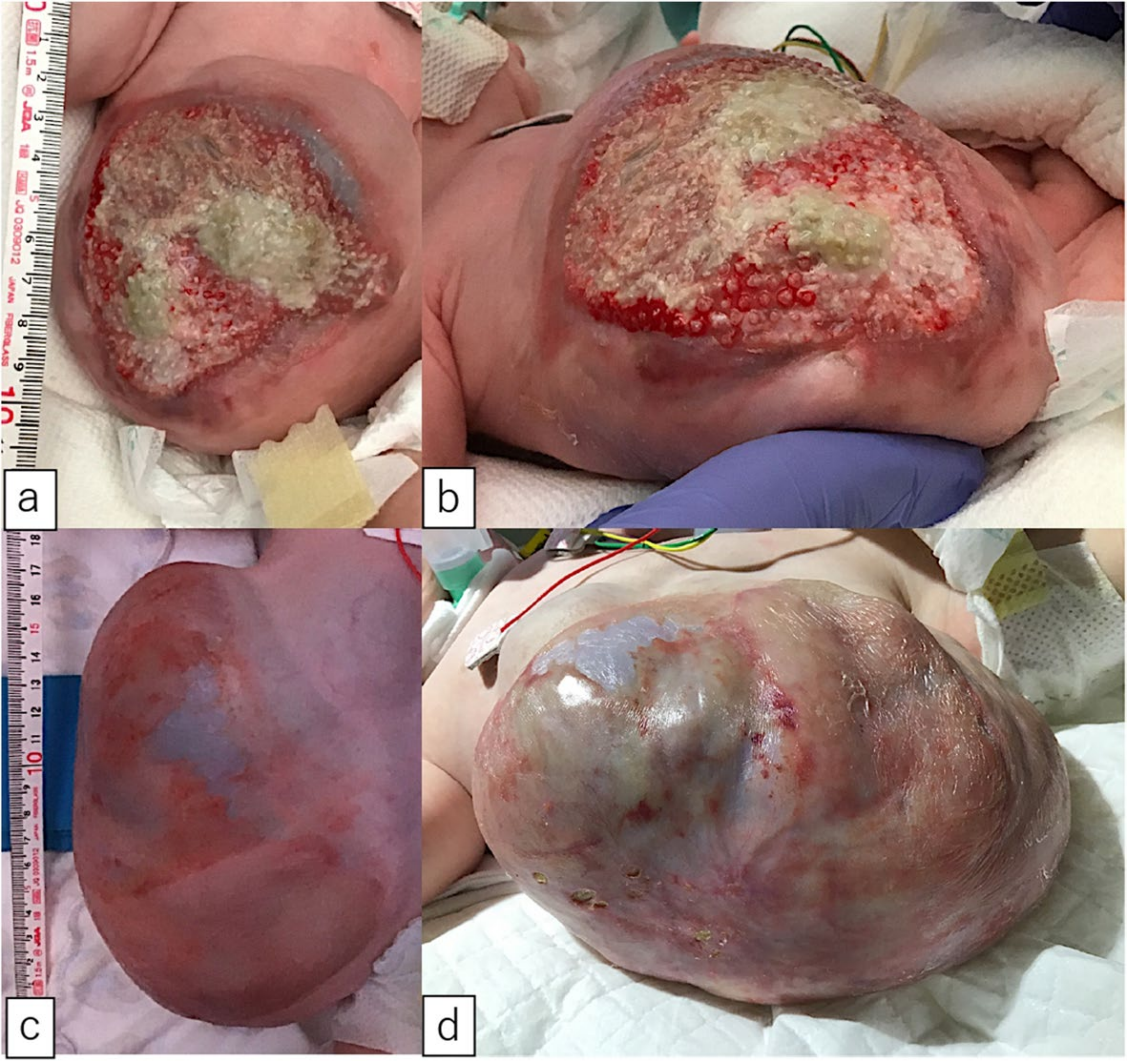
** a**, **b** The defect hole, including the artificial dermis, formed the granulation tissue completely. **c**, **d** Complete epithelialization

**Fig. 6:**
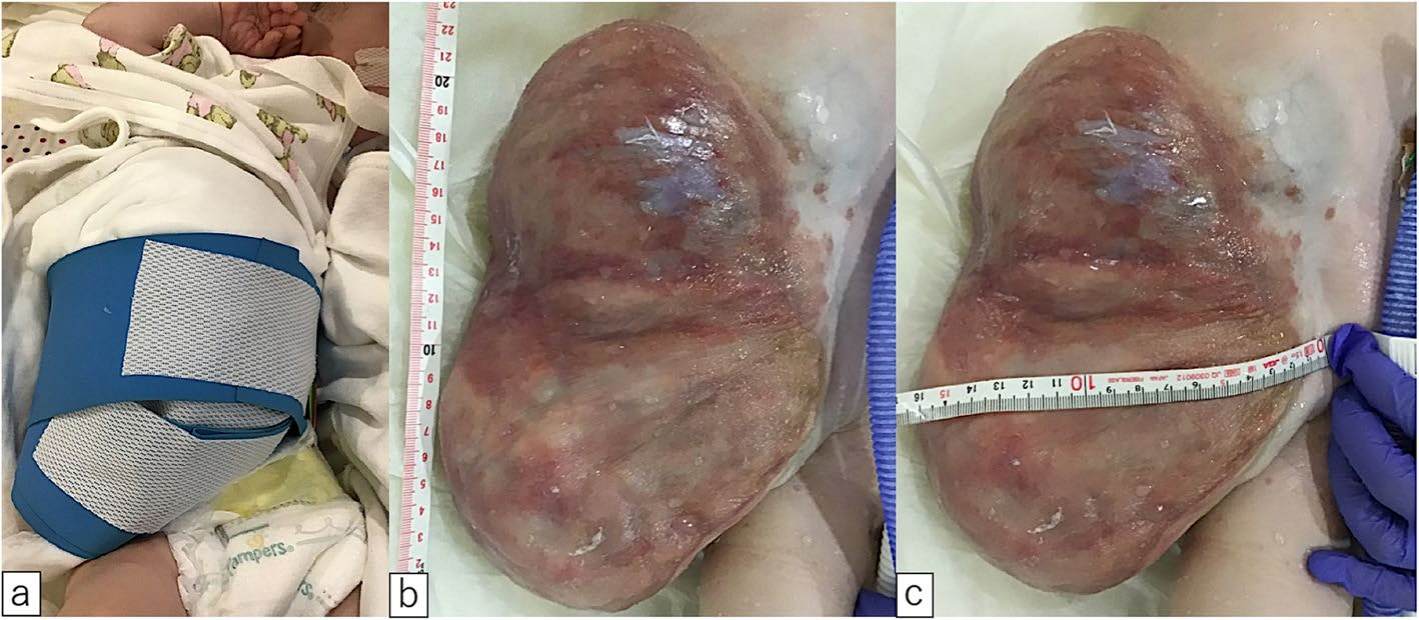
** a** Compression bandage was wrapped at the skin to prevent further skin stretching. **b**, **c** Wound condition at the age of eight months. The skin expansion was improved and prolapsed organs slightly reduced into the abdominal cavity

A male fetus was referred to our hospital with a prenatal diagnosis of a ruptured omphalocele. The patient was born at 38 weeks and 3 days of gestation, weighing 1896 g, via cesarean section. The picture at birth revealed a ruptured giant omphalocele with a defect hole of 10 cm × 10 cm causing herniation of the total liver and intestine (Fig. 1a and c). Because the remaining amniotic membrane could not cover the abdominal wall defect (Fig. 1d) and the wound retractor could not be placed, the patient underwent silo placement using GORE® DUALMESH® Biomaterial (W.L. Gore & Associates G.K., Tokyo, Japan) (Fig. 2a–c). The artificial mesh was plicated from 4 days of age (Fig. 3a). The herniated viscera were gradually reduced into the abdominal cavity; however, the defect size was large; hence, a collagen-based artificial dermis, TERUDERMIS® Artificial Dermis Silicone Membrane Type (ALCARE Co., Ltd., Tokyo, Japan), was patched at the defect hole with a fibrous capsule (Fig. 3b and c) at 19 days of age, which was covered by Gore Dualmesh. After fixing the artificial dermis for 10 days, a fresh and smooth granulation tissue was created at the edge of the artificial dermis (Fig. 3d), and the artificial mesh was removed. The center of the artificial dermis was not completely replaced with granulation tissue, and some collagen dermis was still present. This was found by checking the dermis by inspection and palpation. Inflammatory marker showed increased value with antibiotic therapy, and the wound culture was positive for *Trichophyton* species and *Escherichia coli* at the age of 14 days and 33 days, respectively (Fig. 4). Considering the wound was infected, we adopted NPWTi-d for controlling the wound infection and promoting granulation tissue formation at the remaining dermis at 33 days of age to promote granulation and to control infection. We used a 3 M™ V.A.C.® Ulta Therapy Unit with 3 M™ VeraFlo™ Therapy (KCI, Tokyo, Japan). The negative pressure setting was − 50 mmHg, with an instillation volume of 6 mL, dwell time of 5 min, and cycle length of 2 h. The 3 M™ V.A.C. Veraflo™ Therapy Dressing was changed every 3–4 days. To prevent dermatopathy and organ injuries, hydrocolloid wound dressing and non-adherent wound dressing, Mepitel® One (Monthlycke Health Care, Tokyo, Japan) was applied to cover the skin and wound (Fig. 3e), respectively below the V.A.C. Veraflo™ Therapy Dressing (Fig. 3f). NPWTi-d was removed at 60 days of age when the granulation tissue was well formatted, including the artificial dermis site (Fig. 5a and b). The wound was managed with prostandin ointment and appropriate debridement, resulting in complete epithelialization at 5 months of age (Fig. 5c and d). Epithelialization had successfully completed; however, the abdominal organs extended to the abdominal wall defect, leading to stretching of the skin. To expand the abdominal cavity and prevent further skin stretching, compression bandage was wrapped around the abdomen (Fig. 6a) since 5 months of age. The skin expansion was improved and prolapsed organs had slightly reduced into the abdominal cavity at 8 months of age (Fig. 6b and c). In future, abdominoplasty with excess skin resection and using musculocutaneous flap such as latissimus dorsi when the abdominal cavity has sufficient space for reducing the organs is planned.

## Discussion and conclusions

The standard management strategy for treating giant omphaloceles has not been determined, and various closure methods have been used. Rupture can occur pre- or postnatally and is estimated to be 7–15% [8]. In the case of a ruptured omphalocele, fluid balance and normothermia should be the focus [9] and broad-spectrum prophylactic antibiotics should be administered to prevent infection [9, 10]. Ruptured omphaloceles require immediate intervention. Primary fascial closure is first selected as it obviates morbidity [11]; however, giant omphalocele cases cannot be closed primarily and requires artificial bridges. Our case was a prenatally ruptured giant omphalocele with scoliosis; therefore, a silo with delayed closure was needed because of the low volume of the abdominal cavity.

In this case, three therapeutic steps were adopted. The first was silo placement by the GORE® DUALMESH® Biomaterial. This case was a prenatally ruptured omphalocele with scoliosis; hence, the abdominal cavity had insufficient space. Herniated organs, including the liver and total intestine, should be covered with artificial patches to prevent infection. The silo was gradually plicated; however, the large defect hole and small abdominal cavity prevented the complete reduction of the herniated organs.

The second procedure was artificial dermis placement. Because there was no room for abdominal cavity insulation, and the silo infection prevented granulation tissue formation, the defect hole was covered with TERUDERMIS® artificial dermis silicone membrane. TERUDERMIS® is made of atelocollagen with less antigenicity and reconstructs dermis-like granulation by infiltrating the collagen sponge of the patient’s own cells and capillaries from the wound bed. The silicone membrane layer prevents infection and controls the exudates.

The third procedure was NPWTi-d. NPWTi-d revealed better control of the infected wounds than conventional NPWT. NPWTi-d instills a topical solution (e.g., normal saline) into the wound and is evacuated by negative pressure after a specified interval. NPWTi-d has a greater effect on decreasing the number of bacteria per wound and promoting granulation tissue formation [12–15]. Sepsis is the most frequent complication in giant omphaloceles [8] with a 13% complication rate; therefore, we adopted NPWTi-d, which is a new trial for managing ruptured giant omphaloceles, rather than conventional NPWT. The artificial dermis is vascularized within 2–4 weeks and eventually remodels into a dermal comparison [16, 17]. NPWT can accelerate incorporation of the artificial dermis [18–20]. Histological examination also confirmed that the artificial dermis was completely neovascularized after 5–8 days by NPWT [18]. The manufacturing company recommended 2–3 weeks for engrafting; therefore, the artificial dermis combined with NPWT took a shorter period to cover the defect hole than recommended. In this case, we wanted to perform NPWTi-d synchronously with the artificial dermis. However, because the intestine was directly under the artificial dermis, NPTWi-d might damage and perforate the intestine. Consequently, we decided to perform NPTWi-d after a certain amount of dermal-like granulation had formed in the artificial dermis. Hence, NPTWi-d effectively promoted granulation tissue, covering the herniated organs without major complications.

The appropriate NPWTi-d setting has not been determined in neonates. An international consensus guidelines for NPWTi-d [21] reported that an appropriate range of instillation dwell time is 10 − 20 min with an appropriate volume of instillation solution which visibly saturates the foam. The appropriate negative pressure is −125 to −150 mmHg with a negative pressure time of 1 − 2.5 h. We adopted an instillation volume of 6 mL, dwell time of 5 min, negative pressure setting of −50 mmHg, and a negative pressure time of 2 h. The instillation volume was determined by the volume of solution that completely saturated the foam. The dwell time was 5 min, which was shorter than that mentioned in the guidelines; however, we used normal saline as the instillation solution due to Japanese insurance requirements, unlike the anti-microbial product containing solutions mentioned in the guidelines. We considered the 5 min dwell time as sufficient for irrigation because 10 − 20 min of dwell time, as mentioned in the guidelines, included the wait-time for the anti-microbial effect of the solution. A negative pressure time of 2 h was determined by calculating an approximate mean time from the range of 1 − 2.5 h. Negative pressure was set to the lowest pressure of V.A.C.® Ulta Therapy Unit because although NPWTi-d has gained wide adoption in various areas, negative pressure on the wound can cause complications. Adverse outcomes include bleeding [22, 23], retained sponge in the wound [24, 25], intestine perforation [26–29], and dermatitis/skin maceration [26]. Neonatal tissue is more fragile compared to adult tissue; hence, we attempted to prevent these complications with a low negative pressure, hydrocolloid wound dressing, and wound contact layer. In fact, no significant complications, including dermatitis, occurred in this case.

We adopted NPWTi-d for mild-moderate omphalocele case because we expected that NPWTi-d contributed to reducing the incidence of infection. Prophylactic NPWTi-d has not fully demonstrated effectiveness in preventing infection; however, the effectiveness of NPWTi-d in reducing surgical site infection has recently been reported [30]. NPWTi-d is theoretically effective in preventing infection; hence, routine use of NPWTi-d can contribute to decreasing infection in mild-moderate omphalocele cases.

Artificial membranes followed by artificial dermis were used to promote a fibrous capsule and artificial dermis granulation, which protects against organ damage. In conclusion, NPWTi-d achieved better control of infection and promoted wound healing. NPWTi-d combined with artificial dermis can be an effective treatment for ruptured giant omphaloceles.

## Data Availability

Not applicable.

## Abbreviations

NPWT: Negative-pressure wound therapy
NPWTi-d: Negative-pressure wound therapy with irrigation and dwell time

## References

[1] Khan FA, Hashmi A, Islam S (2019). Insights into embryology and development of omphalocele. Semin Pediatr Surg.

[2] Grosfeld JL, Weber TR (1982). Congenital abdominal wall defects: Gastroschisis and omphalocele. Curr Probl Surg.

[3] Adetayo OA, Aka AA, Ray AO (2012). The use of intra-abdominal tissue expansion for the management of Giant Omphaloceles: review of literature and a Case Report. Ann Plast Surg.

[4] Pandey V, Gangopadhyay AN, Gupta DK, Sharma SP, Kumar V (2014). Non-operative management of giant omphalocele with topical povidone-iodine and powdered antibiotic combination: early experience from a tertiary centre. Pediatr Surg Int.

[5] Aldridge B, Ladd AP, Kepple J, Wingle T, Ring C, Kokoska ER (2016). Negative pressure wound therapy for initial management of giant omphalocele. Am J Surg.

[6] Binet A, Gelas T, Jochault-Ritz S, Noizet O, Bory JP, Lefebvre F (2013). VAC® therapy a therapeutic alternative in giant omphalocele treatment: a multicenter study. J Plast Reconstr Aesthetic Surg.

[7] Kilbride KE, Cooney DR, Custer MD (2006). Vacuum-assisted closure: a new method for treating patients with giant omphalocele. J Pediatr Surg.

[8] Saxena AK, Raicevic M (2018). Predictors of mortality in neonates with giant omphaloceles. Minerva Pediatr.

[9] Gonzalez KW, Chandler NM (2019). Ruptured omphalocele: diagnosis and management. Semin Pediatr Surg.

[10] Åarcã E, Aprodu SG (2014). Past and Present in Omphalocele Treatment in Romania. Chirurgia..

[11] Levy S, Tsao K, Cox CS, Phatak UR, Lally KP, Andrassy RJ (2013). Component separation for complex congenital abdominal wall defects: not just for adults anymore. J Pediatr Surg.

[12] Goss SG, Schwartz JA, Facchin F, Avdagic E, Gendics C, Lantis JC (2012). Nd. Negative pressure Wound Therapy with Instillation (NPWTi) Better reduces post-debridement Bioburden in chronically infected lower extremity wounds than NPWT alone. J Am Coll Clin Wound Spec.

[13] Gabriel A, Shores J, Heinrich C, Baqai W, Kalina S, Sogioka N (2008). Negative pressure wound therapy with instillation: a pilot study describing a new method for treating infected wounds. Int Wound J.

[14] Brinkert D, Ali M, Naud M, Maire N, Trial C, Téot L (2013). Negative pressure wound therapy with saline instillation: 131 patient case series. Int Wound J.

[15] Lessing C, Slack P, Hong KZ, Kilpadi D, McNulty A (2011). Negative pressure wound therapy with controlled saline instillation (NPWTi): Dressing Properties and Granulation response in vivo. Wounds.

[16] Machens HG, Berger AC, Mailaender P (2000). Bioartificial skin. Cells Tissues Organs.

[17] Heimbach DM, Warden GD, Luterman A, Jordan MH, Ozobia N, Ryan CM (2003). Multicenter postapproval clinical trial of Integra dermal regeneration template for burn treatment. J Burn Care Rehabil.

[18] Eo S, Kim Y, Cho S (2011). Vacuum-assisted closure improves the incorporation of artificial dermis in soft tissue defects: Terudermis(®) and Pelnac(®). Int Wound J.

[19] Kim EK, Hong JP (2007). Efficacy of negative pressure therapy to enhance take of 1-stage allodermis and a split-thickness graft. Ann Plast Surg.

[20] Molnar JA, DeFranzo AJ, Hadaegh A, Morykwas MJ, Shen P, Argenta LC (2004). Acceleration of Integra incorporation in complex tissue defects with subatmospheric pressure. Plast Reconstr Surg.

[21] Kim PJ, Attinger CE, Steinberg JS, Evans KK, Lehner B, Willy C (2013). Negative-pressure wound therapy with instillation: international consensus guidelines. Plast Reconstr Surg.

[22] Petzina R, Malmsjö M, Stamm C, Hetzer R (2010). Major complications during negative pressure wound therapy in poststernotomy mediastinitis after cardiac surgery. J Thorac Cardiovasc Surg.

[23] Kiessling AH, Lehmann A, Isgro F, Moritz A (2011). Tremendous bleeding complication after vacuum-assisted sternal closure. J Cardiothorac Surg.

[24] Leijnen M, Steenvoorde P (2008). A retained sponge is a complication of vacuum-assisted closure therapy. Int J Low Extrem Wounds.

[25] Beral D, Adair R, Peckham-Cooper A, Tolan D, Botterill I (2009). Chronic wound sepsis due to retained vacuum assisted closure foam. BMJ.

[26] Rentea RM, Somers KK, Cassidy L, Enters J, Arca MJ (2013). Negative pressure wound therapy in infants and children: a single-institution experience. J Surg Res.

[27] Rao M, Burke D, Finan PJ, Sagar PM (2007). The use of vacuum-assisted closure of abdominal wounds: a word of caution. Colorectal Dis.

[28] Trevelyan SL, Carlson GL (2009). Is TNP in the open abdomen safe and effective?. J Wound Care.

[29] Fischer JE (2008). A cautionary note: the use of vacuum-assisted closure systems in the treatment of gastrointestinal cutaneous fistula may be associated with higher mortality from subsequent fistula development. Am J Surg.

[30] Han Z, Yang C, Wang Q, Wang M, Li X, Zhang C (2021). Continuous negative pressure drainage with intermittent irrigation leaded to a risk reduction of Perineal Surgical site infection following laparoscopic Extralevator Abdominoperineal Excision for low rectal Cancer. Ther Clin Risk Manag.

